# Effects of Drainage on Human Life

**Published:** 1844-06-15

**Authors:** 


### EFFECTS OF DRAINAGE ON HUMAN LIFE.

The Rev. Professor Buckland, at a public meeting held in Oxford last week, said that, in the parish of St. Margaret, Leicester, containing 22,000 inhabitants, it appeared that one portion of it was effectually drained, some parts but partially so, and others not at all. In the latter, the average duration of life is thirteen years and a half, while in the same parish, where the drainage is only partial, the average is twenty-two years and a half, thereby showing the frightful effects of a bad atmosphere.—*Lond. Med. Times.*

